# Unemployment Insurance

**Published:** 1912-11-02

**Authors:** 


### NATIONAL INSURANCE ACT DEPARTMENT.

### XXXIII.—Unemployment Insurance—(*continued*).

In the National Insurance Bill, as originally presented to Parliament, there was a distinction between the rate of benefit to be paid during unemployment to workmen engaged in the building trade or in construction of works on the one hand, and those employed in mechanical engineering, shipbuilding, or construction of vehicles on the other hand. The benefit proposed for the first group was a payment of 6s. a week, while in the latter group the rate was to be 7s. a week. It was, however, felt to be undesirable to make this differentiation, partly because of the difficulty of drawing a hard-and-fast line of demarcation between the respective trades, and also because the data upon which it was sought to justify the different treatment were admittedly imperfect and of comparatively small dimensions. In course of time, as the statistics of the unemployment fund are tabulated and examined, the data for a satisfactory solution will become available.

Should it transpire that the rate of unemployment is materially different in the two classes it will be possible for the Board of Trade to exercise the limited power reserved to it under the Act of revising the rate of benefit for any particular trade. It is thus clear that the principle of the trade basis is preserved not only as regards the present obligatory operation of the Act, but also ultimately with respect to the differentiation of the rates of benefit.

### Statistics of Unemployment.

As a matter of interest it may be pointed out that the available statistics relating to unemployment had been furnished to the Board of Trade by Trade Unions, and from these figures the annual average rates of unemployment over the twenty years 1891 to 1910 inclusive are as follows:—

| Class of Trade Union | Annual Average Rate of Unemployment per Cent. |
|---|---|
| Building (Carpenters and Plumbers only) ... ... | 5.1 |
| Engineering ... ... ... | 5.6 |
| Shipbuilding ... ... ... | 10.4 |
| Coachbuilding ... ... ... | 3.5 |
| Mill-Sawyers ... ... ... | 3.6 |

The particular section of the building trade for which figures are here given appears to enjoy a moderate rate of unemployment, but this is the section that is least affected by seasonal changes; and on the basis of figures supplied to the actuary responsible for the estimates by the Board of Trade, it appeared that on the whole the rate of unemployment in the building trade generally would be twice as much as the figure given in the above table. As, therefore, the number employed in the building trades was estimated to be 1,248,000, or more than 50 per cent. of the total estimate of those who would come within the insured trades, whilst the shipbuilders were estimated at the relatively small number of 137,000, effect was sought to be given to the supposed higher rate of unemployment in the building class by cutting down the rate of benefit as compared with the engineers.

### Rates of Contribution.

We indicated in our last article that the unemployment insurance was of a contributory character and that the contributions were to be apportioned between the workman, the employer, and the State. The normal rate of contribution from a workman in an insured trade is 2½d. for every week he is employed. When, however, the workman is below the age of eighteen the rate of contribution is reduced to 1d. It will be remembered in passing that the rate of benefit below the age of eighteen is also at a lower rate than the normal.

In like manner the normal rate of contribution payable by the employer is 2½d. a week, and a similar reduction of the rate to 1d. is made when the workman is under the age of eighteen. There is, however, a very important provision by which relief is given to the employer who maintains a workman in regular employment throughout the year.

In order to obtain the benefit of this concession application must be made by the employer to the Board of Trade within one month after the termination of a calendar year, or such other period as may be prescribed, and it must be shown to the satisfaction of the Board that he has had a workman in his service continuously throughout the period and has paid on his behalf contributions to the unemployment fund for not less than forty-five weeks, or in the proportion of forty-five to fifty-two weeks if the period is less than one year, as it may be on the first occasion if the Board of Trade decides to make the periods coincide with the calendar year. The amount of the refund to the employer in such circumstances is fixed as one-third of the contributions paid by him on his own behalf in respect of the particular workman during the period.

This is a concession of no mean value, and specific account had to be taken of it, as we shall presently see, in framing the estimates of the rates of contribution for the benefits.

### The Object of the Concession.

It is, however, the object of the concession to give the employer a financial interest in keeping his workmen regularly employed, and by this means to reduce the rate of unemployment and the charge on the unemployment fund. This was one of the conditions laid down by Sir H. Llewellyn Smith as being necessary in a sound scheme. It is open to doubt whether the refund will have the desired effect, for in the extreme case of an employer having paid fifty-two weekly contributions on behalf of a workman in the course of a year the amount of the refund will only be 3s. 7d. With a large number of workmen the value of the concession may be considerable, but it would seem that in influencing continuous employment it must be measured by the individual workman; and thus, while the large employer during periods of active business may receive a substantial reduction in his contributions, it is doubtful whether, in periods of depression, or in trades where workmen have been in the habit of frequently changing their employers, the allowance will have much, if any, effect.

### The Contribution of the State.

Unlike the State contribution in the health insurance part of the Act, which, it will be remembered, is paid as an addition to the benefits and not directly as an addition to the weekly contributions, the aid given to the unemployment fund by the State grant is proportional to the total amount of contributions paid by the employers and workmen jointly. The proportion borne by the State is one-third of the total contributions and it is to be credited to the unemployment fund annually.

In the normal case, therefore, the weekly contribution is made up as follows:—

| | *d.* |
|---|---|
| **From the workman ... ... ... ... ...** | **$2\frac{1}{2}$** |
| **From the employer ... ... ... ... ...** | **$2\frac{1}{2}$** |
| **Together ... ... ... ... ... ...** | **5** |
| **Add one-third as State grant ... ... ...** | **$1\frac{2}{3}$** |
| **Total contribution ... ... ...** | **$6\frac{2}{3}$** |

It will, however, be noticed that, as the State grant is proportional to the amount actually paid in contributions by the employer on his own account and on behalf of the workman, the special concession in the case of workmen regularly employed will have the effect of reducing the State grant also. In such case the weekly contribution will, in effect, be made up as follows:—

| | *d.* |
|---|---|
| **From the workman ... ... ... ... ...** | **$2\frac{1}{2}$** |
| **From the employer ($\frac{2}{3}$ of $2\frac{1}{2}$*d.*) ... ... ...** | **$1\frac{2}{3}$** |
| **Together ... ... ... ... ... ...** | **$4\frac{1}{6}$** |
| **Add one-third as State grant ... ... ...** | **$1\frac{7}{18}$** |
| **Total contribution ... ... ... ...** | **$5\frac{5}{9}$** |

We can now see the full effect of the concession in reducing the total contribution from $6\frac{2}{3}$d. to $5\frac{5}{9}$d., that is a difference of $1\frac{1}{9}$d. a week.

### How the Contributions are Payable.

The contributions are payable by means of special unemployment insurance stamps which have to be affixed to cards in the same manner as for the health insurance. The employer in the first place pays for the stamp, or, in other words, pays the joint contribution for himself and the workman, and subsequently makes the deduction of the workman's contribution from his wage. There is, however, an important distinction between the two systems, for in the health insurance if a stamp has already been affixed to a card in respect of a particular week by the first employer, no further stamp is required to be affixed by any subsequent employer, whilst under the unemployment system each period of employment of less than a week is to count as a week, subject to certain modifications, and thus a stamp has to be affixed by each employer. The modifications here referred to are that if the period of employment does not exceed one day the contribution from the employer and workman is 1d. each, and if more than one day but not exceeding two days, the contribution is 2d. each. The object of this general provision is to discourage casual employment in the insured trades, and a further regulation is made whereby the Board of Trade is enabled to make arrangements with employers in the insured trades so that by obtaining such casual labour as they may require through the Labour Exchanges they will be allowed to pay the contributions on the basis of the number of men employed for the week without reference to the variations in the actual men employed.

